# Persistence of Mast Cell-Positive Synovitis in Early Rheumatoid Arthritis Following Treatment With Conventional Synthetic Disease Modifying Anti-Rheumatic Drugs

**DOI:** 10.3389/fphar.2020.01051

**Published:** 2020-07-14

**Authors:** Felice Rivellese, Francesca W. Rossi, Giovanni Giorli, Filomena Napolitano, Amato de Paulis, Costantino Pitzalis

**Affiliations:** ^1^ Centre for Experimental Medicine & Rheumatology, William Harvey Research Institute, Barts and The London School of Medicine and Dentistry, Queen Mary University of London, London, United Kingdom; ^2^ Department of Translational Medical Sciences (DiSMeT) and Center for Basic and Clinical Immunology Research (CISI), University of Naples Federico II, Naples, Italy

**Keywords:** mast cells, synovitis, synovial membrane, rheumatoid arthritis, inflammation, treatment response

## Abstract

Mast cells (MCs) are immune cells infiltrating the synovial membrane and implicated in the pathogenesis of Rheumatoid Arthritis (RA). Their infiltration in the synovia of early RA patients has been shown to be associated with systemic inflammation, disease activity and autoantibody positivity. Here, we analyzed their presence in matched synovial samples obtained by ultrasound-guided synovial biopsies pre- and post-treatment with conventional synthetic Disease Modifying Anti-Rheumatic Drugs (csDMARDs) (n=20). Upon IHC staining, patients were classified as MC^+ve/-ve^ based on the presence/absence of CD117+ synovial MCs. At baseline, MC^+ve^ patients had significantly higher synovial inflammation, inflammatory markers, disease activity and a higher prevalence of lympho-myeloid aggregates. Synovial biopsies after 6 months of treatment with csDMARDs showed a significant reduction of synovitis scores, but only a partial reduction of MC numbers. Accordingly, 45% of patients (9/20) were MC^+ve^ after treatment, in association with significantly higher degree of synovitis and higher proportion lympho-myeloid aggregates. Finally, significantly lower patients with MC^+ve^ synovitis at 6 months reached Low Disease Activity (LDA), while the association of MCs with disease activity was independent from lymphoid aggregates, after adjustment for BMI and age. Overall, this study confirms the relevance of MCs as part of the inflammatory infiltrate in the synovia of RA patients, warranting further investigations in larger cohorts to clarify their role in disease progression and response to treatment and their relevance as prognostic markers and potential therapeutic targets.

## Introduction

Mast cells (MCs) are tissue-resident cells of the innate immunity, involved in a number of physiological and pathological processes, including infections, cancer, and chronic inflammatory diseases (Voehringer, 2013; Krystel-Whittemore et al., 2016; Varricchi et al., 2017; Varricchi et al., 2018). Mast cells are present in the synovial membrane (SM) in physiological conditions (De Paulis et al., 1996) and have been implicated in various rheumatic diseases (Suurmond et al., 2016), including rheumatoid arthritis (RA) (Rivellese et al., 2017). In fact, synovial MC numbers are significantly increased in inflammatory conditions such as RA (Crisp et al., 1984; Gotis-Graham and McNeil, 1997; Gotis-Graham et al., 1998). However, recent evidences suggest that MC contribution to autoimmune diseases can be complex and multifaceted (Brown and Hatfield, 2012). In the context of RA, for example, human MCs have been shown to induce immunomodulatory effects *in vitro* (Rivellese et al., 2015). Similarly, *in vivo* findings in animal models yielded contrasting results on the contribution of mast cells to the development of arthritis (Lee et al., 2002; Zhou et al., 2007; Pitman et al., 2011). Recent data suggest that their contribution to RA may be different in various disease stages, i.e. essential during the early phases, but dispensable during the late effector phases (Schubert et al., 2015; van der Velden et al., 2016). Thus, despite a substantial amount of data produced over the last years, the role of MCs in RA remains to be clarified (Rivellese et al., 2019b). When considering the well-known heterogeneity of RA (Pitzalis et al., 2013; Smolen et al., 2016; Firestein and McInnes, 2017) and the multifaceted functions of MCs, it is possible to hypothesize that the presence and functions of MCs in synovia may be different in various disease subsets. Recently, the analysis of MCs in the synovia of a large cohort of conventional synthetic disease-modifying anti-rheumatic drugs (csDMARDs)-naïve early RA patients has substantiated such hypothesis, as it showed a strong association of MCs with the infiltration of lymphoid and myeloid cells, which are defining a specific histological subset of patients with a so-called lympho-myeloid pathotype (Pitzalis et al., 2013; Rivellese et al., 2018). Since the lympho-myeloid synovial pathotype has been associated with disease outcomes (Humby et al., 2019), here we aimed to analyze the presence/absence of MCs in synovial biopsies at baseline and 6 months after treatment with csDMARSDs.

## Materials and Methods

### Patient Samples and Ultrasound-Guided Synovial Biopsy

Synovial tissue was obtained by ultrasound-guided synovial biopsy from DMARD-naïve patients with early (<12 months) RA (n=20), enrolled in the Pathobiology of Early Arthritis Cohort (PEAC) cohort of the Centre for Experimental Medicine and Rheumatology of Queen Mary University (London) at Barts Health NHS trust (Kelly et al., 2015). At the baseline visit, following written informed consent, patients underwent synovial biopsy of the most inflamed joint (Synovial thickening >=2). Afterwards, patients started treatment with csDMARDs with a treat-to-target approach, according to a standardized protocol in line with local guidelines [NICE (National Institute for Health and Care Excellence), 2018]. All patients were started on methotrexate—unless contraindicated—in combination of hydroxychloroquine or sulfasalazine. At 6 months, patient had a repeated ultrasound-guided synovial biopsy of the same joint (n=20). An overview of csDMARDs use up to 6 months is presented in **Table 2**. More specifically, two patients were not treated with csDMARDs, two were in monotherapy with Methotrexate, one with hydroxychloroquine, four in combination therapy with methotrexate and hydroxychloroquine, 10 with methotrexate and sulfasalazine, and one with methotrexate, hydroxychloroquine, and sulfasalazine. All patients fulfilled the 2010 EULAR criteria for RA (Aletaha et al., 2010). All procedures were performed following written informed consent and were approved by the hospital’s ethics committee (REC 05/Q0703/198).

### Histological Analyses of Synovial Samples

Synovial sections underwent standard H&E staining and semi-quantitative (SQ) assessment of synovitis according to a previously validated score (Krenn) (Krenn et al., 2006). Sequentially cut sections underwent Immunohistochemical (IHC) staining and upon SQ scoring (0‑4), sections were stratified into synovial pathotypes according to the degree of immune cell infiltration: i) Lymphoid- grade 2/3 B cell aggregates, CD20≥ 2 and/or CD138>2, ii) Myeloid- CD68 SL≥ 2, CD20 ≤ 1 and/or CD3≥1, CD138 ≤ 2, and iii) Fibroid- CD68 SL<2 and CD3, CD20, CD138<1) (Humby et al., 2009; Rivellese et al., 2019a). Following IHC staining for CD117, patients were classified as MC+/MC-, based on the presence/absence of synovial mast cells and MC density (n of cells/mm^2^) was calculated by automated cell counting (cellSens, Olympus).

### Statistical Analyses

Measures of central tendency and dispersions and statistical analyses are indicated in each figure legend. P values of <0.05 were considered statistically significant. For the regression analyses, the glm function from package stats v3.6.2 was used, using DAS28 at 6 months as predicted value and BMI, age, lymphoid aggregates (binary), and MCs (binary) as predictors. The performance of the models without and with MCs were compared by ANOVA. Data have been analysed using R Studio Version 1.2.5033.

## Results

### Mast Cells Are Associated With Defined Histological Features of Synovitis and Severe Disease Activity in Early RA

**Figure 1:**
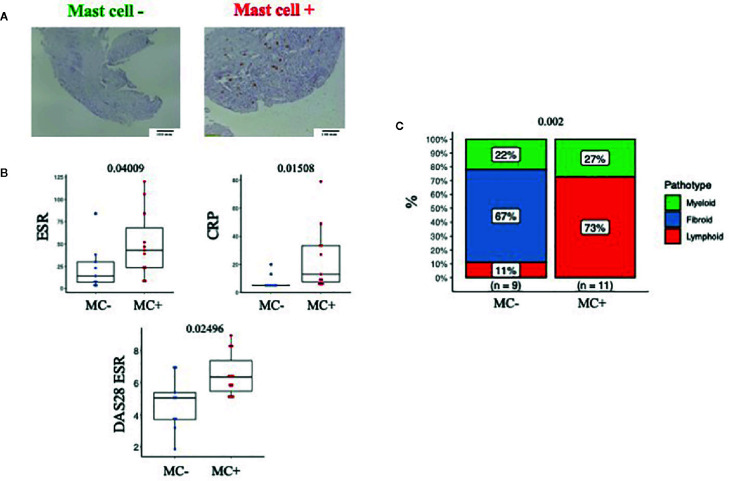
**(A)** Representative example of IHC staining for CD117 (c-kit) and classification of patients into MC^-ve^ (left) and MC^+ve^ (right). Line at 100um. **(B)** ESR, CRP and DAS28 in MC^-ve^ and MC^+ve^ patients. n=20 patients, pre-treatment synovial biopsy. ESR, Erythrocyte Sedimentation Rate; CRP, C Reactive Protein; DAS28, Disease activity score 28 joints. **(C)** Pathotype distribution in MC^-ve^ and MC^+ve^ patients.Mann Whitney test in B, Fisher in C, p value is displayed when significant (<0.05), ns, non-significant (>0.05).

First, we explored whether the presence of MCs in synovia could identify early RA patients with a severe phenotype. To this aim, we classified patients into MC^+ve^ and MC^-ve^ based on the presence/absence of synovial MCs. Representative images of this classification are shown in **Figure 1A**. MC^+ve^ patients (9/20, 45%) had significantly higher levels of inflammatory markers (ESR and CRP) and disease activity (DAS28) compared to MC^-ve^ (**Figure 1B** and **Table 1**). Overall, this indicates that the presence of MCs in synovia identifies patients with higher levels of systemic inflammation and severe disease. Accordingly, MC^+ve^ patients had significantly higher synovitis scores and a higher prevalence of the lympho-myeloid pathotype (**Table 1** and **Figure 1C**). However, there were no significant differences in DAS28, in the delta change of DAS28 from baseline and the prevalence of low disease activity in patients stratified as MC- or MC+ at baseline (**Table 1**).

**Table 1:** Baseline features in patients stratified according to Mast cell presence.

	Overall	MC negative	MC positive	P value
	(N=20)	(N=9)	(N=11)	
**Age**				0.3215
Mean (SD)	52.9 (18.7)	47.0 (20.9)	57.3 (16.5)	
Median [Min, Max]	53.0 [23.0, 81.0]	45.5 [23.0, 79.0]	60.0 [27.0, 81.0]	
**Disease onset**				1
Mean (SD)	4.61 (2.48)	4.29 (1.70)	4.82 (2.93)	
Median [Min, Max]	4.00 [2.00, 12.0]	5.00 [2.00, 7.00]	4.00 [2.00, 12.0]	
**ESR**				0.0400
Mean (SD)	38.3 (34.9)	23.4 (25.8)	50.5 (37.7)	
Median [Min, Max]	27.5 [2.00, 120]	14.0 [2.00, 84.0]	43.0 [8.00, 120]	
**CRP**				0.0150
Mean (SD)	16.9 (19.3)	7.56 (5.36)	24.5 (23.3)	
Median [Min, Max]	7.50 [5.00, 79.0]	5.00 [5.00, 20.0]	13.0 [5.00, 79.0]	
**ACPA positive, %**	60.00%	55.60%	63.60%	0.6499
**RF positive, %**	50.00%	44.40%	54.50%	0.6562
**DAS28 ESR**				0.0249
Mean (SD)	5.69 (1.75)	4.67 (1.69)	6.52 (1.36)	
Median [Min, Max]	5.59 [1.88, 8.92]	5.05 [1.88, 7.00]	6.34 [5.09, 8.92]	
**Tender joint count**				0.1184
Mean (SD)	12.1 (8.91)	8.67 (8.43)	14.9 (8.64)	
Median [Min, Max]	11.5 [1.00, 28.0]	5.00 [2.00, 27.0]	12.0 [1.00, 28.0]	
**Swollen Joint Count**				0.1809
Mean (SD)	8.00 (7.09)	5.67 (4.47)	9.91 (8.40)	
Median [Min, Max]	6.50 [1.00, 26.0]	4.00 [1.00, 16.0]	8.00 [2.00, 26.0]	
**VAS global health**				0.0200
Mean (SD)	67.3 (28.5)	50.9 (31.4)	80.6 (17.7)	
Median [Min, Max]	77.5 [2.00, 100]	48.0 [2.00, 100]	79.0 [48.0, 100]	
**Joint biopsied**				n.a.
Knee	1 (5.0%)	0 (0%)	1 (9.1%)	
MCP	1 (5.0%)	1 (11.1%)	0 (0%)	
PIP	1 (5.0%)	0 (0%)	1 (9.1%)	
Wrist	17 (85.0%)	8 (88.9%)	9 (81.8%)	
**Synovitis scores**				0.0311
No synovitis (0-2)	8 (40.0%)	6 (66.7%)	2 (18.2%)	
Low synovitis (3-5)	4 (20.0%)	2 (22.2%)	2 (18.2%)	
High Synovitis (6-9)	8 (40.0%)	1 (11.1%)	7 (63.6%)	
**Pathotypes**				
Fibroid	6 (30.0%)	6 (66.7%)	0 (0%)	0.0019
Lymphoid	9 (45.0%)	1 (11.1%)	8 (72.7%)	
Myeloid	5 (25.0%)	2 (22.2%)	3 (27.3%)	

ES, RErythrocyte Sedimentation Rate; CRP, C Reactive Protein; ACPA, Anti Citrullinated Protein Antibodies, RF, Rheumatoid Factor; DAS28, Disease Activity Score 28 joints; VAS,Visuoanalog scale.

### Treatment With csDMARDs Induced a Significant Reduction of Synovial Inflammation and a Partial Reduction of Mast Cell Numbers

**Figure 2:**
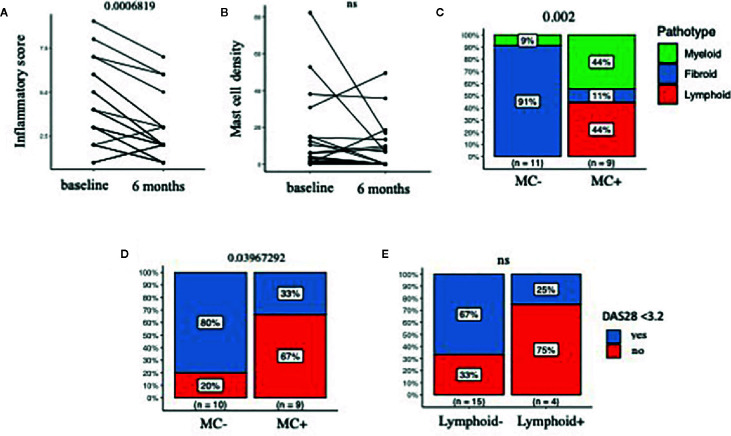
**(A, B)** Synovitis score (Krenn score) in A and MC density (number/mm2) in B, in matched ultrasound-guided biopsies at baseline and 6 months after treatment with csDMARDs. **(C)** Distribution of pathotypes at 6 months in MC^-ve^ and MC^+ve^ patients. **(D, E)** Prevalence of low disease activity (LDA), defined as DAS28<3.2, at 6 months in in MC^-ve^ and MC^+ve^ patients (D) and lymphoid-ve and lymphoid+ve patients (E); N=20 patients, pre- and post-treatment biopsy in A-B, post-treatment in C-E. MC, Mast cells; DAS28, Disease activity score 28 joints; csDMARDs, conventional synthetic Disease Modifying Anti-Rheumatic Drugs; paired samples Wilcoxon test in A and B, Fisher exact test in C-D-E. P values are displayed when significant (<0.05), ns, non-significant (>0.05).

Having demonstrated that the presence of MCs in synovia identifies early RA patients with severe disease, we next looked at the effect of csDMARDs treatment on synovial inflammation, by analyzing repeated synovial biopsies at 6 months. We observed a significant reduction of the synovitis score (Krenn score, **Figure 2A**) and only a partial non-significant reduction of MC numbers (**Figure 2B**). Accordingly, synovial MCs were present in 45% of patients (MC^+ve^ 9/20) at 6 months, in association with higher synovitis scores and a higher prevalence of lympho-myeloid aggregates (**Figure 2C** and **Table 2**). This shows that treatment with csDMARDs has an impact on synovial inflammation, as it induces a significant reduction of synovitis. Nonetheless, almost half of the patients have non-resolving synovial inflammation, with MC infiltration accompanied by lympho-myeloid aggregates.

**Table 2:** Six months outcomes in patients stratified according to MC presence at baseline and 6 months.

	BASELINE BIOPSY			
**6 months outcomes**	Overall	MC negative	MC positive	
	**(N=20)**	**(N=9)**	**(N=11)**	
**DAS28ESR**				0.1518
Mean (SD)	2.41 (1.03)	3.80 (2.01)	3.21 (1.78)	
Median [Min, Max]	2.08 [1.36, 3.83]	3.50 [1.46, 7.56]	3.03 [1.36, 7.56]	
**Delta DAS28ESR**				0.4326
Mean (SD)	2.51 (1.32)	2.21 (1.66)	2.73 (1.03)	
Median [Min, Max]	2.22 [0.160, 5.21]	2.07 [0.160, 5.21]	2.22 [1.36, 4.89]	
**DAS28 <3.2**	55.00%	66.70%	45.50%	0.1977
**Methotrexate, %**	85.00%	66.70%	100%	0.1637
**csDMARDs numbers**				0.2669
0	2 (10.0%)	2 (22.2%)	0 (0%)	
1	3 (15.0%)	1 (11.1%)	2 (18.2%)	
2	14 (70.0%)	5 (55.6%)	9 (81.8%)	
3	1 (5.0%)	1 (11.1%)	0 (0%)	
**Steroids, %**	12 (60.0%)	5 (55.6%)	7 (63.6%)	1
	**6 MONTHS BIOPSY**			
	**Overall**	**MC negative**	**MC positive**	
	**(N=20)**	**(N=11)**	**(N=9)**	
**ESR**				0.3627
Mean (SD)	15.7 (22.5)	8.20 (5.77)	24.0 (30.8)	
Median [Min, Max]	8.00 [2.00, 95.0]	7.50 [2.00, 19.0]	8.00 [2.00, 95.0]	
**CRP**				0.5383
Mean (SD)	6.06 (2.67)	6.00 (3.16)	6.13 (2.10)	
Median [Min, Max]	5.00 [5.00, 15.0]	5.00 [5.00, 15.0]	5.00 [5.00, 10.0]	
**DAS28ESR**				0.0788
Mean (SD)	3.21 (1.78)	2.40 (0.919)	4.11 (2.10)	
Median [Min, Max]	3.03 [1.36, 7.56]	2.09 [1.36, 3.83]	3.64 [1.46, 7.56]	
**Tender Joint Count**				0.0325
Mean (SD)	5.20 (7.34)	2.27 (4.15)	8.78 (8.96)	
Median [Min, Max]	2.00 [0, 22.0]	0 [0, 14.0]	6.00 [0, 22.0]	
**Swollen Joint Count**				0.09357
Mean (SD)	2.40 (2.93)	1.27 (1.74)	3.78 (3.56)	
Median [Min, Max]	1.00 [0, 10.0]	1.00 [0, 5.00]	3.00 [0, 10.0]	
**VAS globa health**				0.2234
Mean (SD)	29.7 (29.1)	21.5 (22.9)	39.7 (34.0)	
Median [Min, Max]	19.5 [0, 89.0]	12.0 [0, 70.0]	34.0 [0, 89.0]	
**Delta DAS28ESR**				1
Mean (SD)	2.51 (1.32)	2.45 (1.56)	2.57 (1.08)	
Median [Min, Max]	2.22 [0.160, 5.21]	2.78 [0.160, 5.21]	2.14 [1.36, 4.89]	
**DAS28V3 <3.2**	55.00%	72.70%	33.30%	0.0396
**Methotrexate, %**	85.00%	72.70%	100%	0.4736
**csDMARDs number**				0.4687
0	2 (10.0%)	2 (18.2%)	0 (0%)	
1	3 (15.0%)	2 (18.2%)	1 (11.1%)	
2	14 (70.0%)	6 (54.5%)	8 (88.9%)	
3	1 (5.0%)	1 (9.1%)	0 (0%)	
**Steroids, %**	12 (60.0%)	5 (45.5%)	7 (77.8%)	0.1968
**Synovitis scores**				0.0045
No synovitis (0-2)	12 (60.0%)	10 (90.9%)	2 (22.2%)	
Low synovitis (3-5)	4 (20.0%)	1 (9.1%)	3 (33.3%)	
High Synovitis (6-9)	4 (20.0%)	0 (0%)	4 (44.4%)	
**Pathotypes**				0.0009
Fibroid	11 (55.0%)	10 (90.9%)	1 (11.1%)	
Myeloid	5 (25.0%)	1 (9.1%)	4 (44.4%)	
Lymphoid	4 (20.0%)	0 (0%)	4 (44.4%)	

DAS28, Disease Activity Score 28 joints; csDMARDs, conventional synthetic Disease Modifying Anti-Rheumatic Drugs; ESR, Erythrocyte Sedimentation Rate; CRP, C Reactive Protein; ACPA, Anti Citrullinated Protein Antibodies, RF, Rheumatoid FactorVAS, Visuoanalog scale.

p, Fisher exact test or Mann-Whitney, as appropriate.

### Persistence of Mast Cells at 6 Months Was Associated With a Higher Disease Activity and a Lower Remission Rate

In parallel to non-resolving synovial inflammation, the presence of MCs after 6 months of treatment with synthetic DMARDs was associated with numerically higher DAS28 values (p=0.07) and significantly higher tender joint count (p=0.03) and significantly lower rates of Low Disease Activity (LDA), as defined by DAS28 <3.2 (**Table 2** and **Figure 2D**). As shown in **Table 2**, most of patients were treated with a combination of two csDMARDs, without statistically significant differences between MC+ ad MC- groups.

When patients were stratified according to the presence/absence of lympho-myeloid aggregates at 6 months, we found no significant differences in the prevalence of low disease activity (**Figure 2E**) nor in any other clinical parameter at 6 months (data not shown).

To further confirm the association of MCs with disease activity at 6 months, we used multiple linear regression to predict 6 months DAS28, using the presence/absence of MCs and lymphoid aggregates as predictors, after correcting for age and BMI. Interestingly, the presence of lymphoid aggregates per se did not improve the prediction model, while the presence of MCs significantly improved its performance (ANOVA p value 0.00657 when comparing the full model including MCs, lymphoid aggregates, age and BMI to the model with lymphoid aggregates, age and BMI). The results of the full regression model with age, BMI, lymphoid aggregates and MCs are shown in **Table 3**.

**Table 3:** Multiple linear regression for 6 months DAS28.

Term	Estimate	Std error	Statistic	p.value	95% CI	
(Intercept)	-1.74	2.91	-0.59	0.56	-7.46	3.97
Mast cell presence	3.04	1.12	2.71	0.02	0.84	5.24
Lymphoid aggregates	-2.26	1.24	-1.81	0.10	-4.71	0.18
Age	0.03	0.02	1.53	0.15	-0.01	0.08
Body Mass Index	0.0	0.08	0.94	0.37	-0.08	0.23

Although the small numbers should be taken into account when interpreting these results, these analyses suggests that the presence of MCs at 6 months biopsy is associated with disease severity, independently of BMI, age and lymphoid aggregates.

## Discussion

We here report the analysis of mast cells in the synovia of patients with early untreated Rheumatoid Arthritis undergoing ultrasound-guided synovial biopsies before and after treatment with csDMARDs. Our results indicate that synovial MCs are associated with disease severity at baseline and non-resolving MC^+ve^ synovitis after treatment with csDMARDs is associated with a lower response to csDMARDs.

The results at baseline are in agreement with previous publications (Gotis-Graham and McNeil, 1997; Gotis-Graham et al., 1998), including our recent observations showing that stratification of patients according to the abundancy of synovial MCs in early untreated RA identifies patients with severe disease (Rivellese et al., 2018).

Here, we classified patients based on the simple presence/absence of synovial MCs, rather than into groups based on the relative abundancy of mast cells. Thus, our results indicate that the simple presence of MCs in the synovia of patients with early untreated RA, independently from the degree of infiltration, is able to identify patients with higher disease severity at baseline.

Additionally, we assessed MCs in matched ultrasound guided synovial biopsies following treatment with csDMARDs, showing a significant reduction of the synovitis score and a partial reduction of MC density. Accordingly, 45% of the patients were classified as MC^+ve^ in the repeated post-treatment biopsy, in association with a lower prevalence of low disease activity. In other words, non-resolving MC^+ve^ synovitis is associated with a reduced response to csDMARDs, as only 33% of MC+ patients reach a low disease activity, in comparison to 80% of MC negative.

To our knowledge, one previous work reported the analysis of MCs in post-treatment synovial biopsies, that failed to identify a clear pattern in the effects of treatment on synovial MC infiltration, most likely because of the lack of treatment standardization and the very low number of patients (n=6) (Gotis-Graham et al., 1998). In our manuscript, we analysed matched post-treatment synovial biopsies from an observational cohort, thus in the absence of randomization, however patients were treated according to a standardized protocol in line with treat-to-target local guidelines and tight follow-up, with visits at 1 month, 3 months, and 6 months [NICE (National Institute for Health and Care Excellence), 2018]. Accordingly, most patients were treated with a combination of two csDMARDs, with no differences in MC+ and MC- groups, suggesting that the lack of response in MC^+ve^ patients is not associated with sub-optimal treatment.

This observation is in line with a recent publication describing significantly higher numbers of synovial mast cells and B cells in patients who did not maintain remission after 1 year, which suggest that synovial MCs could be used as predictors of disease flare (Ramírez et al., 2016).

Importantly, since the presence of MCs in synovia is strongly associated with lympho-myeloid cells, it could be argued that the association of MCs with treatment outcomes is indirect. Indeed, we have previously shown that high MC in synovia are associated with the infiltration of lympho-myeloid cells, both *ex vivo* in early untreated RA patients and *in vivo* in animal models of antigen induced arthritis, and MCs were able to activate B cells inducing the production of ACPA autoantibodies (Rivellese et al., 2018). Thus, it could be hypothesized that lympho-myeloid cells are the real culprit in defining response/non-response to treatment. However, when patients in our cohort were classified as lympho-myeloid positive/negative, based on the presence/absence of lympho-myeloid aggregates at 6 months, we did not observe significant differences in the number of patients reaching a low disease activity. Because of the small numbers, we can’t exclude a type 2 error, thus we cannot exclude the association of lymphoid aggregates with treatment response. However, regression analyses suggested MC presence at 6 months is an independent predictor of disease activity, independently of lymphoid aggregates, after correction for BMI and age.

Overall, these observations are in line with our recent manuscript, showing that histologically defined lympho-myeloid patients are associated with worse disease activity at baseline and higher levels of radiographic progression at 12 months, but pathotypes at baseline do not associate with response to csDMARDs. On the contrary, reduction of lymphoid related genes assessed by molecular analysis of pre and post-treatment biopsies was associated with treatment response (Humby et al., 2019). However, also when considering the relatively low numbers of patients included in our current study, the relevance of other immune cells in addition to MCs can’t be excluded, since it is possible that with a larger cohort other significant differences will emerge. At the same time, the identification of significant differences in such a small cohort points to the relevance of MCs, particularly when considering that their simple presence in synovia, independently from the degree of infiltration, is able to identify patients with higher disease severity. Nonetheless, additional work in larger cohorts is essential to confirm our observations and dissect the diverse contribution of MCs and other immune cells as markers of disease severity, progression and response to treatment. In fact, although a few study have looked at synovial membrane factors as predictors of response to csDMARDs (Vieira-Sousa, 2011) and at the effect of treatment on synovitis (Haringman, 2005), none to our knowledge include the analysis of MCs. In recent years, the attention has switched to biologic treatment, and a number of publications described both histological and molecular signatures that can predict treatment response to biologics (Dennis et al., 2014; Aterido et al., 2019). However, to date, the relevance of synovial MCs in relation to response to conventional or biologic DMARDs has not been explored.

In conclusion, in a small observational cohort of early RA patients with matched pre and post treatment synovial biopsies, we describe higher disease activity in association with the presence of synovial MCs at baseline and lower response to csDMARDs in patients with persistence of MC infiltration. Although additional studies in larger cohorts are needed to dissect the diverse contribution of mast cells and other immune cells, these results suggest that the analysis of synovial MCs contributes to the definition of the synovial inflammatory landscape.

## Data Availability Statement

The datasets generated for this study are available on request to the corresponding author.

## Ethics Statement

The studies involving human participants were reviewed and approved by Queen Mary University – REC 05/Q0703/198. The patients/participants provided their written informed consent to participate in this study.

## Author Contributions

FR: study design, experiments, data acquisition, data analysis, manuscript preparation and revision. FWR: data analysis, manuscript preparation and revision. GG: data analysis, manuscript revision. FN: data analysis, manuscript revision. AP: interpretation of experimental results, manuscript revision. CP: study design, interpretation of experimental results, manuscript revision. FR wrote the manuscript. All authors contributed to the article and approved the submitted version.

## Funding

The Pathobiology of Early Arthritis Cohort (PEAC) was supported by the MRC (grant G0800648). FR is supported by an NIHR Transitional Research Fellowship (TRF-2018-11-ST2-002). Versus Arthritis provided funding infrastructure support (Grant code 20022). The views expressed are those of the author(s) and not necessarily those of the MRC, Versus Arthritis, NHS, the NIHR or the Department of Health and Social Care.

## Conflict of Interest

The authors declare that the research was conducted in the absence of any commercial or financial relationships that could be construed as a potential conflict of interest.
